# Nonlinear HbA1c thresholds reveal accelerated atherogenic remodeling and improved risk reclassification in type 2 diabetes

**DOI:** 10.3389/fcvm.2026.1751404

**Published:** 2026-01-30

**Authors:** Vehbi Şirikçi, Hüseyin Avni Fındıklı

**Affiliations:** Internal Medicine Department, Necip Fazil City Hospital, Kahramanmaras, Türkiye

**Keywords:** atherogenic dyslipidemia, atherogenic index of plasma, lipid remodeling, triglyceride-to-HDL cholesterol ratio, type 2 diabetes mellitus

## Abstract

Dysglycemia, lipid metabolism, and cardiovascular disease (CVD) progression in type 2 diabetes (T2D) are closely interconnected, yet the non-linear lipid remodeling processes underlying atherogenic dyslipidemia remain insufficiently defined. This study aimed to identify HbA1c thresholds associated with accelerated lipid-driven atherogenesis, quantify the mediating role of the triglyceride-to-HDL cholesterol ratio (TG/HDL-C)—a surrogate of insulin-resistance–related lipid metabolism—and assess the incremental predictive value of the Atherogenic Index of Plasma (AIP) within the clinically ambiguous “glycemic gray zone.” A total of 271 adults with T2D not receiving lipid-lowering therapy were retrospectively grouped by HbA1c: good (<7.0%), moderate (7.0%–8.49%), and poor (≥8.5%) control. Atherogenic lipid burden was evaluated using AIP, Castelli indices, TG/HDL-C, non-HDL cholesterol, and remnant cholesterol. Restricted cubic splines were used to explore non-linear HbA1c–lipid relationships; mediation analysis estimated the TG/HDL-C contribution to the HbA1c–AIP pathway; and Net Reclassification Improvement (*N*RI) tested the added predictive value of AIP over conventional lipid markers. All atherogenic indices worsened with deteriorating glycemia (*p* < 0.001). Non-linear inflection points were observed at HbA1c 8.0% for TG/HDL-C and 8.5% for AIP (p_non-linearity < 0.01). TG/HDL-C mediated 56.9% of the HbA1c effect on AIP, indicating its central role in linking hyperglycemia to lipid remodeling. Adding AIP improved cardiovascular risk reclassification, particularly in the 8.0%–8.5% transition range (categorical NRI = 0.384; 95% CI: 0.184–0.584). These findings identify 8.0%–8.5% as a metabolically vulnerable HbA1c threshold marked by accelerated atherogenic dyslipidemia. AIP functions as a sensitive lipid-based marker for cardiometabolic risk detection within this gray zone, while TG/HDL-C acts as a key mechanistic mediator, supporting the integration of atherogenic lipid indices into individualized risk assessment and precision lipid management strategies in T2D.

## 1 Introduction

Type 2 diabetes (T2D) is a rapidly escalating global health problem and a leading contributor to cardiovascular disease (CVD) morbidity and mortality (1). While hyperglycemia has long been recognized as a principal driver of diabetic complications, the mechanistic interplay between deteriorating glycemic control and accelerated atherogenic lipid remodeling remains only partially understood (2). Most prior studies examined linear associations between HbA1c and traditional lipid markers, including LDL-C, HDL-C, and triglycerides (3). Yet diabetic dyslipidemia entails more complex qualitative abnormalities in lipoprotein metabolism—specifically, rising triglyceride-rich lipoproteins, a shift toward small dense LDL, and impaired HDL functionality—that are not fully reflected by standard lipid metrics (4, 5). Composite indices such as the Atherogenic Index of Plasma (AIP) and the triglyceride-to-HDL cholesterol ratio (TG/HDL-C) better capture this atherogenic milieu (6), but how these indices accelerate with worsening dysglycemia, particularly in a non-linear fashion, remains insufficiently quantified.

This gap creates tangible uncertainty in clinical practice, particularly concerning lipid management. Current glycemic targets are largely guided by microvascular outcomes (7, 8), with limited evidence addressing the non-linear escalation in cardiometabolic risk and associated lipoprotein derangements accompanying incremental HbA1c elevations. The HbA1c interval of approximately 8.0%–8.5% represents a therapeutically ambiguous “glycemic gray zone,” in which the pace of cardiovascular risk progression driven by atherogenic dyslipidemia and the need for intensified lipid management remain unclear (9, 10). Consequently, clinical decision-making within this range often lacks mechanistic precision.

Accordingly, this study sought to investigate the interaction between glycemic severity and atherogenic lipid remodeling in T2D. Specifically, we aimed to: (i) define non-linear inflection points across multiple atherogenic indices to identify critical thresholds for accelerated lipid metabolism disturbance; (ii) quantify the mediating role of the TG/HDL-C axis as a key pathway in this relationship; and (iii) evaluate the incremental predictive value of AIP using Net Reclassification Improvement (NRI), with emphasis on the gray-zone interval. By integrating these analytical components, this study aims to enhance early detection of lipid-driven cardiometabolic risk and refine mechanism-informed therapeutic strategies for individuals with T2D.

## 2 Methods

### 2.1 Study design and participants

This retrospective, cross-sectional study was designed to evaluate the association between glycemic control status and lipid-centric atherogenic indices in adults with T2D. Individuals aged 18–65 years with a confirmed diagnosis of T2D who were not taking lipid-lowering medications and who attended the Internal Medicine Outpatient Clinics of Kahramanmaraş Necip Fazıl City Hospital and its Yörükselim affiliated unit between January 1 and September 1, 2025, were screened through the hospital information system (HIS). This lipid-lowering naïve cohort allowed for the assessment of the natural relationship between glycemia and atherogenic lipid profiles.

Patients were included if they had same-day measurements of glycated hemoglobin (HbA1c) and a complete lipid panel, comprising triglycerides (TG), high-density lipoprotein cholesterol (HDL-C), low-density lipoprotein cholesterol (LDL-C), and total cholesterol (TC). Exclusion criteria were defined as the presence of type 1 diabetes, gestational or secondary diabetes, malignancy, coronary artery disease, heart failure, acute or chronic kidney disease, decompensated liver disease, or use of any lipid-lowering agents. Pregnant women, those with thyroid dysfunction (hypo- or hyperthyroidism), and those with incomplete medical records were also excluded, to minimize potential confounders of lipid metabolism.

### 2.2 Data collection and variables

All variables were retrospectively retrieved from the electronic hospital information system (HIS), with no additional patient contact or intervention. Demographic data included age and sex, while glycemic status was determined by HbA1c levels. Lipid-driven atherogenic burden was quantified using core lipid parameters and derived composite indices, calculated as follows: Atherogenic Index of Plasma (AIP) = log_10_(TG/HDL-C), Castelli Risk Index I = TC/HDL-C, Castelli Index II = LDL-C/HDL-C, TG/HDL-C ratio, non-HDL cholesterol = TC—HDL-C, and remnant cholesterol = TC—(LDL-C + HDL-C). These indices were selected to comprehensively assess lipoprotein metabolism and its derangements.

### 2.3 Glycemic categorization

Participants were categorized into three predefined glycemic control groups based on HbA1c thresholds: good control (<7.0%), moderate control (7.0%–8.49%), and poor control (≥8.5%). These cutoffs align with international diabetes guidelines that recommend <7.0% as the general therapeutic target for microvascular protection 7, while levels ≥8.5% are widely recognized as indicating inadequate control requiring treatment escalation 9. The moderate 7.0%–8.49% range represents a clinically uncertain “decision zone,” where the intensification of lipid-driven cardiovascular risk and treatment benefit–risk balance remain controversial 8–10. Therefore, this tripartite categorization enables capturing potential non-linear shifts in lipid metabolism and atherogenic risk that may emerge in this transitional interval. All statistical analyses were conducted using these categories for between-group comparisons.

### 2.4 Ethical approval

The study protocol adhered to the Declaration of Helsinki and received approval from the Kahramanmaraş Sütçü İmam University Ethics Committee (KSÜ-TAREK; protocol number:299/10.08.25). All data were anonymized prior to analysis.

### 2.5 Statistical analysis

All statistical analyses were performed using R software (version 4.3.2; R Foundation for Statistical Computing, Vienna, Austria) with the following packages: tidyverse v2.0.0, car v3.1-2, performance v0.10.8, rms v6.7-0, mediation v4.5.0, and nricens v1.6. The distribution of continuous variables was evaluated using the Shapiro–Wilk test. Normally distributed data were presented as mean ± standard deviation, while skewed data were expressed as median (IQR). Categorical variables were summarized as counts (percentages). Between-group differences were assessed using the Kruskal–Wallis test, followed by Bonferroni-adjusted Mann–Whitney *U* tests for *post hoc* comparisons when overall significance was observed (adjusted *p* < 0.0167). Categorical variables were compared using the chi-square test.

Associations between HbA1c and atherogenic indices were examined using univariable and multivariable linear regression analyses. Univariable models included only atherogenic indices, excluding standard lipid parameters to avoid collinearity, as these are inherent components of the derived indices. Multivariable models were adjusted for age and sex. Right-skewed variables (TG/HDL-C ratio and remnant cholesterol) were log₁₀-transformed prior to analysis. Model assumptions were verified, and no multicollinearity was detected (all VIF <1.1). Model performance was summarized using adjusted R^2^.

To explore the potential non-linear nature of lipid–glycemia relationships, restricted cubic spline regression models with four knots were applied to assess the association between HbA1c and each atherogenic index. Departure from linearity was evaluated using likelihood ratio tests comparing spline-based vs. linear models (*p* < 0.05). Points of maximal curvature along the spline functions were interpreted as metabolic thresholds indicating accelerated atherogenic dyslipidemia. All spline models were adjusted for age and sex.

The mediating role of the TG/HDL-C ratio in the relationship between HbA1c and atherogenic indices was assessed using bias-corrected bootstrap resampling (5,000 iterations). This analysis tested the hypothesis that TG/HDL-C serves as a key mechanistic pathway linking hyperglycemia to adverse lipid remodeling. Indirect effects were considered statistically significant when the corresponding 95% confidence interval did not include zero. Mediation proportions were reported.

To assess the incremental clinical contribution of the Atherogenic Index of Plasma (AIP) to glycemic control classification, Net Reclassification Improvement (NRI) analysis was performed. The baseline logistic regression model included HbA1c, age, and sex, while the updated model additionally incorporated AIP. AIP was entered in its native logarithmic form [AI*P* = log_10_(TG/HDL-C)]. A clinically relevant HbA1c cutoff of 8.5% was applied, and the transitional range between 8.0% and 8.5% was specifically examined to assess the added discriminative value of AIP within the identified metabolic transition zone. Both categorical and continuous NRI values were estimated using bias-corrected bootstrap resampling (5,000 iterations). NRI values were considered statistically significant when the 95% confidence interval did not cross zero.

A two-tailed *p* < 0.05 was considered statistically significant for all analyses.

## 3 Results

### 3.1 Cohort characteristics demonstrate balanced baseline demographics

This retrospective cross-sectional analysis included a total of 271 adults with type 2 diabetes mellitus (T2D). The median age of the cohort was 48.0 years (IQR: 39.0–59.0), and the majority were female (63.1%, *n* = 171). Participants were stratified into three glycemic control categories based on HbA1c levels: good control (*n* = 89, 32.8%), moderate control (*n* = 96, 35.4%), and poor control (*n* = 86, 31.7%). Baseline demographic and routine laboratory characteristics—including age, sex, serum creatinine, alanine aminotransferase (ALT), white blood cell count, hemoglobin, and platelet count—did not differ significantly among the three glycemic control groups (all *p* > 0.05, Table 1).

**Table 1:** Comparative analysis of demographic, laboratory, and atherogenic profiles across HbA1c control groups.

Parameter	Good control (*n* = 89)	Moderate control (*n* = 96)	Poor control (*n* = 86)	*p*-value
Demographic				
Female, *n* (%)	60 (67.4)	62 (64.6)	49 (57.0)	0.337
Age (years)	48.0 (37.0–58.0)	48.0 (39.0–57.0)	48.5 (41.0–59.0)	0.522
Routine laboratory parameters				
Cr (mg/dL)	0.81 (0.70–0.89)	0.80 (0.70–0.89)	0.80 (0.69–0.97)	0.685
ALT (U/L)	21 (16.0–33.0)	24 (18.0–36.0)	25 (17.0–37.0)	0.472
WBC (10^9^ /L)	7.8 (6.58–8.9)	8.2 (7.08–9.6)	8.66 (7.06–10.00)	0.114
Hb (g/dL)	13.3 (12.0–15.1)	14.1 (13.0–15.3)	13.9 (12.7–15.4)	0.278
PLT (10^3^ /μL)	270 (242–313)	277 (239–328)	283 (230–336)	0.419
Lipid Profile				
TC (mg/dL)	191 (159–215)^a^^,^^b^	207 (178–234)^a^	215 (193–247)^b^	**<0**.**001**
LDL-C (mg/dL)	115 (94–131)^a^	125 (102–144)	128.5 (113.0–150.0)^b^	**0**.**009**
HDL-C (mg/dL)	51 (45.0–57.0)	50 (43.0–57.0)	44.0 (37.0–55.0)	**<0**.**001**
TG (mg/dL)	100 (80.0–152.0)^a^^,^^b^	154 (121–196)^a^^,^^c^	213 (146–318)^b^^,^^c^	**<0**.**001**
N-HDL-C (mg/dL)	134 (118.0–158.0)^a^^,^^b^	156 (128–182)^a^	173 (150–200)^b^	**<0**.**001**
R- Chol (mg/dL)	20 (16.0–30.4)^a^^,^^b^	31 (24.1–39.2)^a^^,^^c^	42 (29.3–63.5)^b^^,^^c^	**<0**.**001**
Atherogenic Indices				
AIP	0.33 (0.16–0.50)^a^^,^^b^	0.50 (0.37–0.60)^a^^,^^c^	0.67 (0.52–0.88)^b^^,^^c^	**<0**.**001**
TG/HDL-C ratio	2.15 (1.45–3.13)^a^^,^^b^	3.16 (2.33–3.98)^a^^,^^c^	4.70 (3.31–7.52)^b^^,^^c^	**<0**.**001**
Castelli Index I	3.79 (3.04–4.15)^a^^,^^b^	4.12 (3.68–4.75)^a^	4.85 (4.31–5.97)^b^	**<0**.**001**
Castelli Index II	2.27 (1.81–2.70)^a^^,^^b^	2.50 (2.05–2.93)^a^	2.85 (2.48–3.44)^b^	**<0**.**001**

*Group comparisons were performed using Kruskal–Wallis test for continuous variables and Chi-square test for categorical variables. *post-hoc* pairwise analyses were conducted with Bonferroni-corrected Mann–Whitney *U* tests. Superscript letters indicate significant differences: ^a^Good vs Moderate; ^b^Good vs Poor; ^c^Moderate vs Poor (*p* < 0.05).**ALT, alanine aminotransferase; WBC, white blood cells; Hb, hemoglobin; PLT, platelets; LDL-C, low-density lipoprotein cholesterol; HDL-C, high-density lipoprotein cholesterol; TG, triglycerides; N-HDL-C, non-HDL cholesterol; R-Chol, remnant cholesterol; AIP, atherogenic index of plasma*.

Bold values indicate statistical significance at *p* < 0.05.

### 3.2 Conventional lipid parameters worsen with deteriorating glycemic control

As detailed in Table 1, conventional lipid parameters demonstrated a progressive deterioration across worsening glycemic control categories. Total cholesterol levels differed significantly among groups (*p* < 0.001). *post-hoc* analyses confirmed lower concentrations in the good-control group compared to both the moderate (*p* = 0.008) and poor-control groups (*p* < 0.001), and in the moderate-control vs. the poor-control group (*p* = 0.023).

A similar pattern was observed for LDL-cholesterol (*p* = 0.009 across groups), with the good-control group showing significantly lower levels than both the moderate (*p* = 0.041) and poor-control groups (*p* = 0.003). In contrast, HDL-cholesterol levels were markedly lower specifically in the poor-control group compared to both the good (*p* < 0.001) and moderate-control groups (*p* = 0.009).

Most notably, triglycerides exhibited a pronounced stepwise increase with deteriorating glycemic control (*p* < 0.001 across groups). Levels were significantly lower in the good-control group compared to both the moderate and poor-control groups (each *p* < 0.001), and were also lower in the moderate-control group compared to the poor-control group (*p* = 0.007).

### 3.3 Atherogenic indices show strong, graded associations with glycemic control

Atherogenic burden, quantified through composite indices, demonstrated a clear, graded increase across worsening HbA1c categories (Table 1). Remnant cholesterol, the Atherogenic Index of Plasma (AIP), and the TG/HDL-C ratio all differed significantly among groups (all *p* < 0.001), with pairwise comparisons confirming consistent stepwise elevations from good to moderate to poor control (e.g., remnant cholesterol: *p* = 0.005; AIP: *p* = 0.005; TG/HDL-C: *p* = 0.004). Castelli Risk Indices I and II also varied significantly (both *p* < 0.001); however, unlike the other indices, they showed no further increase between the moderate and poor-control groups (*p* > 0.05), suggesting a plateau effect in response to advanced dysglycemia.

### 3.4 Linear regression identifies AIP as the most sensitive glycemia-linked atherogenic marker

Univariable linear regression revealed strong positive associations between HbA1c and all atherogenic indices (all *p* < 0.001; Table 2). Among these, AIP demonstrated the most robust linear relationship, with each 1% increase in HbA1c corresponding to a 0.058-unit rise in AIP (*β* = 0.058, 95% CI: 0.050–0.066), explaining the largest proportion of variance (*R*^2^ = 0.34). The TG/HDL-C ratio (*β* = 0.35, 95% CI: 0.30–0.40; *R*^2^ = 0.29) and remnant cholesterol (*β* = 0.26, 95% CI: 0.22–0.30; *R*^2^ = 0.24) also exhibited significant dose–response gradients. These associations remained independently significant after adjustment for age and sex, with HbA1c persisting as a major determinant of AIP (*β* = 0.055, 95% CI: 0.047–0.063; *R*^2^adj = 0.32) and the other indices (Table 2).

**Table 2:** Univariable and multivariable linear regression analyses of HbA1c with atherogenic indices.

Variable	Univariate		Multivariate		Adjusted *R*^2^†
	β	95% CI/*p*	β	95% CI/*p*	Univ → Mult
AIP	0.058	(0.05–0.07)/<0.001	0.055	(0.05–0.06)/<0.001	0.34 → 0.32
TG/HDL	0.35	(0.30–0.40)/<0.001	0.33	(0.28–0.38)/<0.001	0.29 → 0.27
R- Chol	0.26	(0.22–0.30)/<0.001	0.24	(0.20–0.28)/<0.001	0.24 → 0.22
Castelli- I	0.19	(0.14–0.24)/<0.001	0.17	(0.12–0.22)/<0.001	0.21 → 0.19
Castelli- II	0.12	(0.08–0.16)/<0.001	0.10	(0.06–0.14)/<0.001	0.18 → 0.16
Non-HDL	7.24	(5.15–9.33)/<0.001	6.95	(4.86–9.04)/<0.001	0.14 → 0.12

*Multivariable models were adjusted for age and sex. β coefficients represent the change in each atherogenic index corresponding to a 1% increase in HbA1c. Log10 transformations were applied to right-skewed variables (TG/HDL-C ratio and remnant cholesterol) to meet linear regression assumptions. All models demonstrated normally distributed residuals and homoscedasticity. Variance inflation factors (VIF) for all independent variables were below 1.1, indicating no multicollinearity. **AIP, atherogenic index of plasma; TG/HDL, triglyceride-to-high-density lipoprotein cholesterol ratio; R-Chol, remnant cholesterol*.

Collectively, these findings establish a robust, independent, and proportional link between deteriorating glycemic control and progressive atherogenic lipid remodeling. AIP emerged as the most responsive index, capturing the largest share of HbA1c-related variance, followed closely by the TG/HDL-C ratio. In contrast, the plateauing of Castelli indices suggests limited sensitivity at higher glycemic levels, underscoring the superior discriminatory capacity of AIP and TG/HDL-C for identifying lipid-driven cardiometabolic risk across the full glycemic spectrum in type 2 diabetes.

### 3.5 Identification of a critical HbA1c transition zone for accelerated atherogenic risk

**Figure 1:**
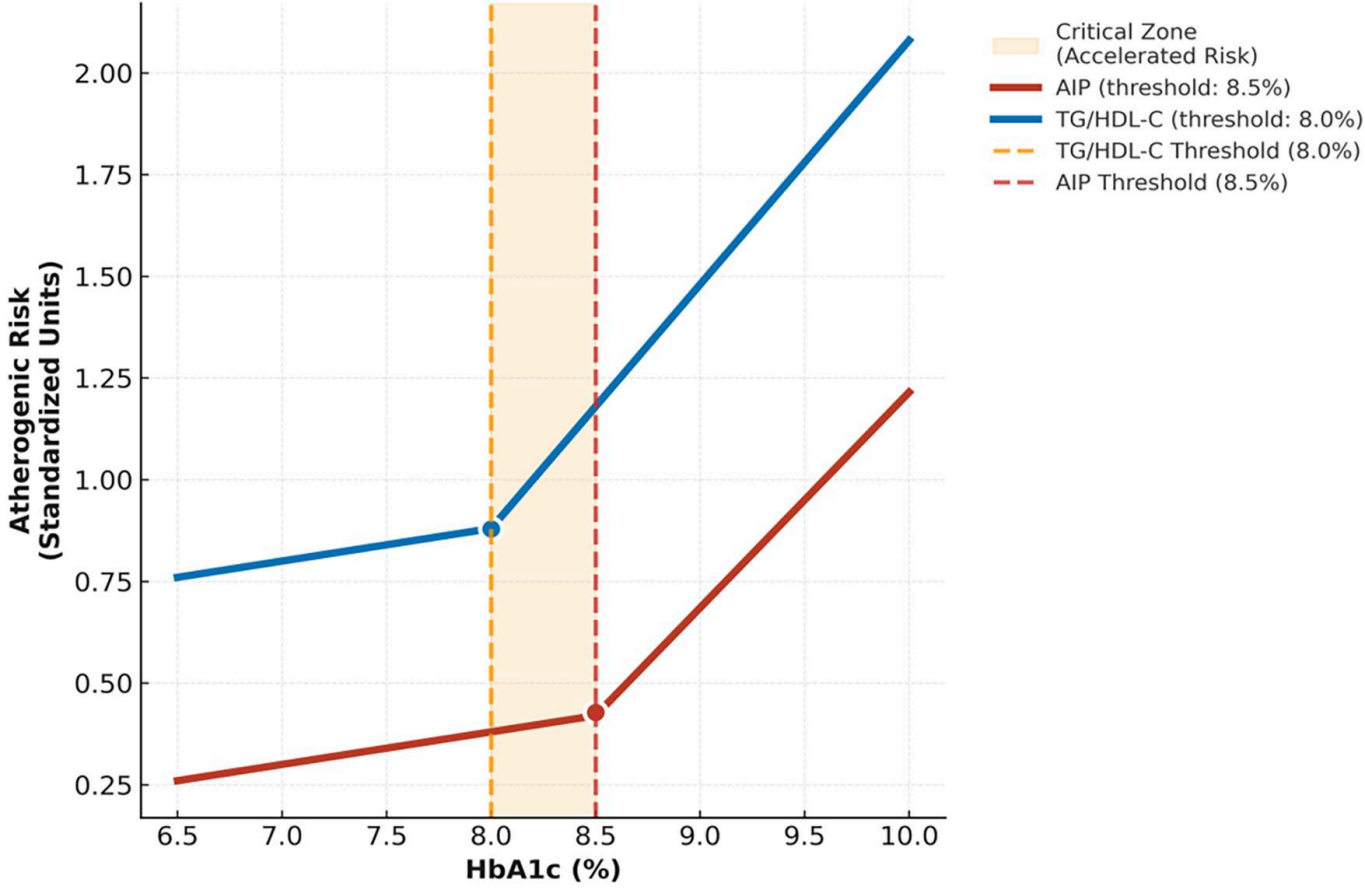
Disproportionate escalation of atherogenic risk beyond HbA1c 8.0%–8.5%: A Mechanistic Inflection Point in T2D. Dose–response curves illustrating glycemic thresholds for accelerated deterioration in atherogenic indices. The triglyceride-to-HDL cholesterol (TG/HDL-C) ratio exhibits an early inflection at 8.0% HbA1c, while the atherogenic index of plasma (AIP) accelerates beyond 8.5%. The intervening “critical zone” (8.0%–8.5%) marks a mechanistic transition from proportional to disproportionate worsening of atherogenic dyslipidemia, predominantly driven by elevations in triglyceride-rich remnants (TRR). These findings emphasize the clinical importance of maintaining HbA1c below 8.0% to prevent crossing the lipid remodeling inflection threshold.

Restricted cubic spline analyses revealed significant non-linear relationships between HbA1c and atherogenic indices, identifying specific thresholds for accelerated lipid deterioration (Table 3; Figure 1). The earliest and most pronounced inflection point was observed for the TG/HDL-C ratio at an HbA1c of 8.0% (p_non-linearity = 0.0021), beyond which its rate of increase accelerated by 52%. For the Atherogenic Index of Plasma (AIP), a critical threshold emerged at HbA1c 8.5%, accompanied by a 45% escalation in slope (p_non-linearity = 0.0012). In contrast, the Castelli indices displayed later and more modest inflection points near HbA1c 9.0%, underscoring their limited sensitivity in detecting early, dysglycemia-driven lipid remodeling. Collectively, these findings define the HbA1c 8.0%–8.5% interval as a pivotal metabolic transition zone characterized by a sharp acceleration in atherogenic dyslipidemia.

**Table 3:** Threshold effects and Non-linear relationships between HbA1c and atherogenic indices.

Atherogenic index	Optimal HbA1c threshold (%)	*ΔR*^2^ (Increase)	Non-linear *p*-value	Slope acceleration (%)
AIP	8.5	+0.035	0.0012	+45
TG/HDL	8.0	+0.032	0.0021	+52
R-Chol	8.5	+0.028	0.0058	+38
Castelli-I	9.0	+0.024	0.0123	+28
Castelli-II	9.0	+0.021	0.0189	+25

*Non-linear associations between HbA1c and atherogenic indices were modeled using restricted cubic splines with four knots. TG/HDL-C ratio and remnant cholesterol were log10-transformed due to right-skewed distributions. Non-linearity was confirmed by likelihood ratio tests (*p* < 0.05), and HbA1c thresholds reflect spline-derived inflection points. Slope acceleration denotes the increased rate of atherogenic worsening above these thresholds. All models were adjusted for age and sex, with residuals meeting normality and homoscedasticity assumptions.**AIP, atherogenic ındex of plasma; TG/HDL, triglyceride-to-high-density lipoprotein cholesterol ratio; R-Chol, remnant cholesterol*.

### 3.6 AIP significantly enhances risk stratification within the metabolic transition zone

**Figure 2:**
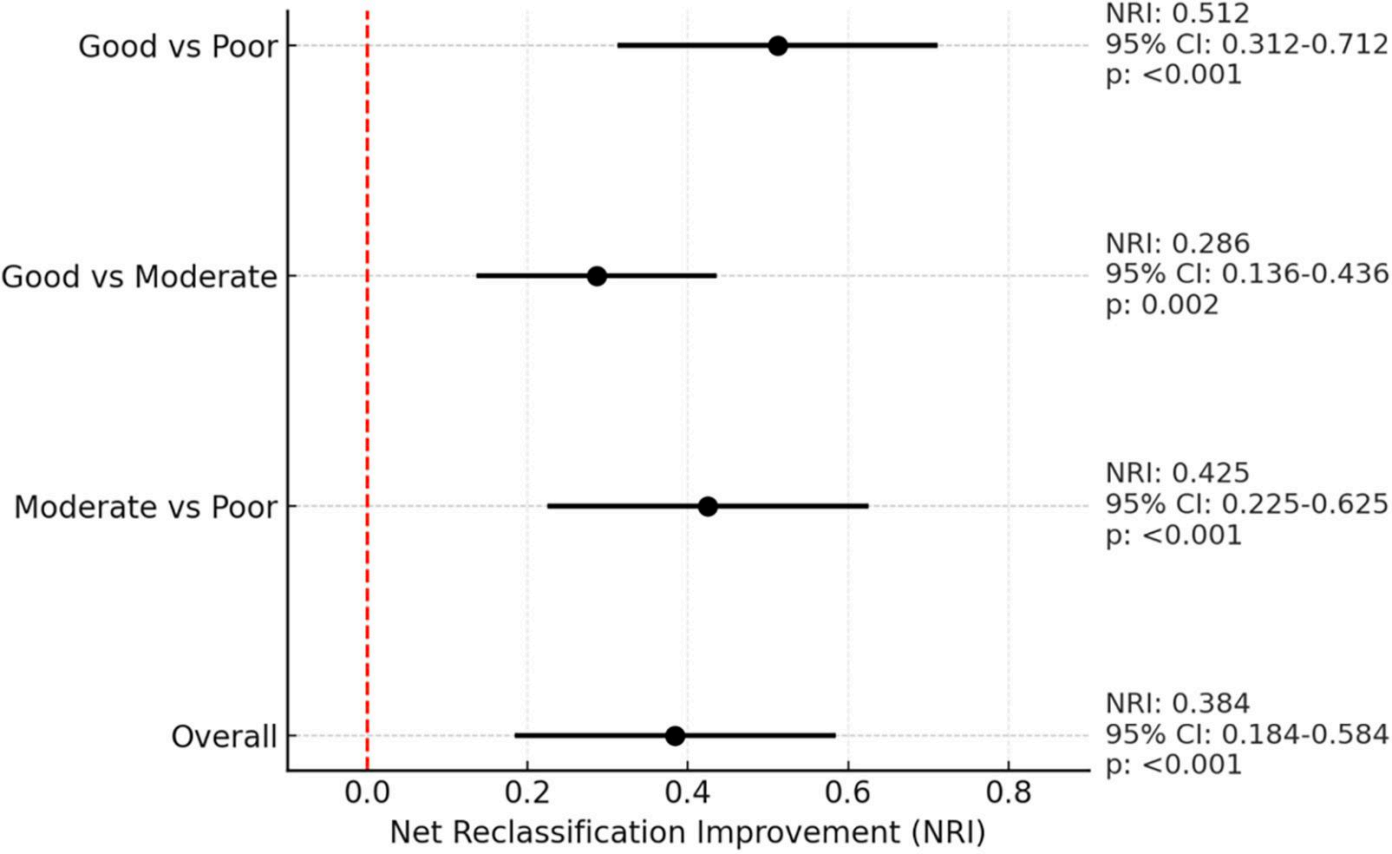
Atherogenic transition zone revealed: AIP sharpens cardiometabolic risk stratification beyond glycemic control. Forest plot illustrates the Net Reclassification Improvement (NRI) achieved by adding the Atherogenic Index of Plasma (AIP) to a baseline model containing HbA1c, age, and sex. Points represent NRI estimates and horizontal lines denote 95% confidence intervals computed using bias-corrected bootstrap resampling (5,000 iterations). The dashed vertical line marks the null effect (NRI=0). The most meaningful enhancement in discrimination is observed within the 8.0%–8.5% HbA1c transition zone, consistent with spline-derived inflection points and reinforcing the clinical relevance of mechanism-based risk stratification.

To translate these mechanistic insights into clinical utility, Net Reclassification Improvement (NRI) analysis was conducted by integrating AIP into a baseline model of HbA1c, age, and sex. The addition of AIP significantly improved the classification of poor glycemic control risk (Figure 2). For the overall cohort, the categorical NRI was 0.384 (95% CI: 0.184–0.584; *p* < 0.001), corresponding to a net 38% improvement in correct reclassification. Notably, this improvement was most prominent within the HbA1c 8.0%–8.5% transition zone precisely aligning with the spline-defined metabolic threshold—thereby underscoring the clinical value of AIP for refining cardiometabolic risk assessment in this glycemically ambiguous range.

### 3.7 TG/HDL-C ratio as the principal mediator of atherogenic lipid remodeling

**Figure 3:**
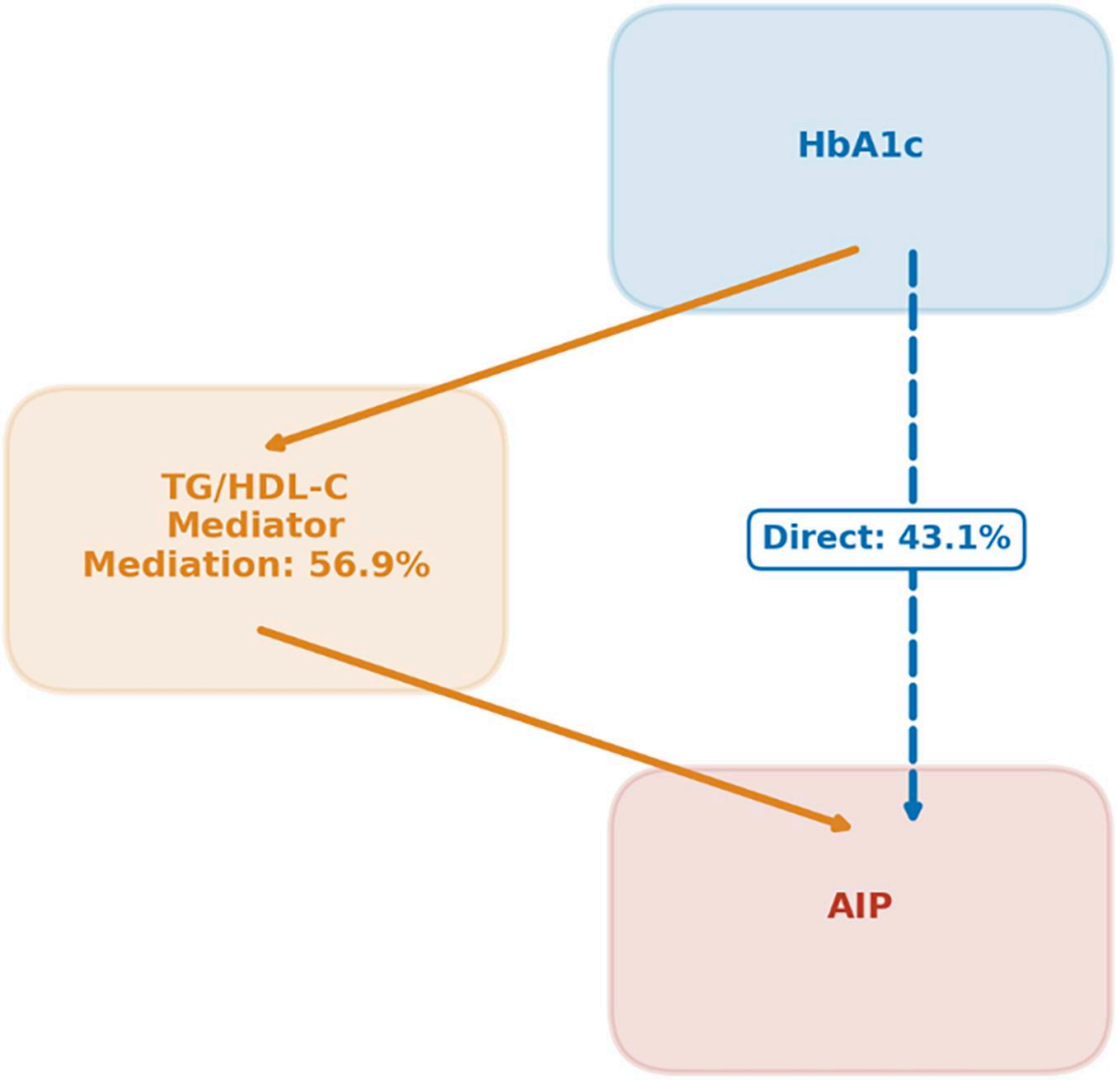
Conceptual clinical framework for mechanism-based atherogenic risk stratification in type 2 diabetes. Mechanistic mediation pathway illustrating the role of TG/HDL-C imbalance in the relationship between hyperglycemia and atherogenic lipoprotein remodeling. The TG/HDL-C ratio accounted for 56.9% of the total effect of HbA1c on AIP, while 43.1% was direct, confirming TG/HDL-C dysregulation as the principal conduit linking glycemic deterioration to exacerbated atherogenic burden.

To elucidate the mechanistic pathway linking dysglycemia to atherogenic lipid remodeling, bias-corrected bootstrap mediation analyses were performed (Table 4). The TG/HDL-C ratio exhibited significant indirect effects across all atherogenic indices (all *p* < 0.001), indicating that hypertriglyceridemia accompanied by HDL-C depletion constitutes a key intermediary disturbance driving the progression from poor glycemic control to adverse lipid remodeling. Notably, 56.9% of the total effect of HbA1c on AIP was mediated through the TG/HDL-C ratio (indirect effect: 0.033; 95% CI: 0.027–0.039), establishing this ratio as the dominant biochemical conduit through which hyperglycemia promotes atherogenic lipoprotein transformation in type 2 diabetes. These findings position TG/HDL-C not only as a sensitive indicator of early metabolic deterioration but also as a mechanistically anchored therapeutic target for disrupting diabetes-related acceleration in cardiovascular risk. The conceptual framework of this mediation pathway is illustrated in Figure 3.

**Table 4:** This analysis quantifies the extent to which the relationship between glycemic burden and atherogenic lipid remodeling is mechanistically explained by TG/HDL-C.

Atherogenic index	Mediation ratio	Indirect effect	95% CI	*p*-value
AIP	56.9%	0.033	(0.027–0.039)	<0.001
TG/HDL	100.0%	0.350	(0.300–0.400)	<0.001
R- Chol	50.0%	0.130	(0.102–0.158)	<0.001
Castelli-I	36.8%	0.070	(0.048–0.092)	<0.001
Castelli-II	29.2%	0.035	(0.021–0.049)	<0.001

*Mediation analysis was performed following the Baron and Kenny framework, with bias-corrected bootstrap resampling (5,000 iterations) to estimate 95% confidence intervals. The intermediary role of TG/HDL-C ratio in the association between HbA1c and atherogenic indices was assessed. Indirect effects were considered statistically significant when confidence intervals did not include zero. **AIP, atherogenic index of plasma; TG/HDL, triglyceride-to-high-density lipoprotein cholesterol ratio; R-Chol, remnant cholesterol*.

## 4 Discussion

The association between glycemic control and cardiovascular disease (CVD) risk in type 2 diabetes (T2D) is well established; however, the dynamic trajectory and critical inflection points of atherogenic lipid remodeling that underlie this relationship remain incompletely characterized (11, 12). Previous studies have primarily focused on linear associations or simple group comparisons, lacking a mechanistic framework that delineates how—and at which glycemic thresholds—lipid-driven cardiovascular risk accelerates as dysglycemia worsens (13). Addressing this knowledge gap, the present study provides novel insights by demonstrating three principal findings: (i) a non-linear trajectory of atherogenic risk with a metabolically vulnerable transition zone between HbA1c 8.0%–8.5%; (ii) a statistically and clinically significant improvement in cardiometabolic risk classification conferred by composite indices—particularly the Atherogenic Index of Plasma (AIP)—as evidenced by Net Reclassification Improvement analysis; and (iii) the identification of the triglyceride-to-HDL cholesterol ratio (TG/HDL-C) as the predominant mechanistic mediator linking hyperglycemia to adverse lipid remodeling.

Consistent with prior evidence, we observed a progressive deterioration across all atherogenic indices with worsening glycemic control. Hyperglycemia-related increases in triglyceride-rich remnant lipoproteins are well documented (14), and a strong positive association between HbA1c and the TG/HDL-C ratio has been previously reported—for example, in Korean populations by Kang et al. (15). However, the present findings extend these observations by demonstrating that this relationship is not merely incremental but instead exhibits a quantifiable acceleration threshold within the HbA1c range of 8.0%–8.5%. The distinct upward inflection in the AIP trajectory likely represents a metabolic tipping point at which intensified insulin resistance, hepatic VLDL overproduction, and reduced lipoprotein lipase activity collectively surpass compensatory capacity (16, 17), triggering a phase of accelerated atherogenic lipoprotein remodeling. Thus, our study not only reaffirms the established link between dysglycemia and atherogenic burden but also introduces a mechanistic framework supporting its threshold-dependent and non-linear behavior (18). These insights offer a compelling biological rationale for proactively re-evaluating cardiovascular risk and intensifying lipid-lowering strategies in patients whose HbA1c lies within this metabolically vulnerable range.

Discrepancies remain in the literature regarding the precise relationship between glycemic control and atherogenic indices. For instance, Jin et al. (19) reported heterogeneous associations across different populations—a finding that contrasts with the distinct and quantifiable threshold phenomenon identified in our cohort. These inconsistencies may stem from population-specific characteristics or, more importantly, from methodological limitations. In particular, the predominant use of linear modeling in previous studies likely obscured non-linear inflection points, thereby underestimating the dynamic nature of lipid–glycemia interactions. Furthermore, while Ma et al. (20) demonstrated the predictive value of AIP and TG/HDL-C for cardiovascular risk, our findings advance this line of evidence by elucidating the mechanistic pathway—specifically, by quantifying the dominant mediating role of TG/HDL-C and thereby establishing the causal link between hyperglycemia and atherogenic lipoprotein remodeling.

The potent mediating role of the TG/HDL-C ratio is firmly grounded in the established pathophysiology of diabetic dyslipidemia. Chronic hyperglycemia and insulin resistance act as dual drivers, stimulating hepatic VLDL overproduction (16) while simultaneously suppressing lipoprotein lipase (LPL) activity. This combination impairs the clearance of triglyceride-rich lipoproteins (TRLs), leading to their accumulation in plasma. As highlighted by Garvey et al. (21), this TRL excess promotes cholesteryl ester transfer protein (CETP)-mediated lipid exchange, generating triglyceride-enriched, cholesterol-depleted HDL particles that are rapidly cleared, thereby lowering HDL-C levels and impairing HDL functionality. This cascade, recognized as highly atherogenic (22), is directly captured by the TG/HDL-C ratio. Consequently, our finding that this ratio mediates 56.9% of HbA1c's effect on atherogenic remodeling positions it not merely as a biomarker but as a central, quantifiable nexus in the causal pathway linking dysglycemia to accelerated cardiovascular risk in T2D.

The clinical relevance of these findings is immediately evident in the context of contemporary therapeutic dilemmas. Following the ACCORD trial (9), the benefit–risk balance of intensive glycemic control in T2D patients with elevated cardiovascular risk has been rigorously re-evaluated. In alignment with this paradigm shift, the AACE/ACE guidelines (10) advocate for individualized glycemic targets and endorse more flexible HbA1c goals for selected patient subgroups. Our results introduce an objective, lipid-based framework to navigate this “clinical gray zone.” Specifically, assessing the Atherogenic Index of Plasma (AIP) in patients with HbA1c levels between 8.0% and 8.5% provides actionable insight that extends beyond glycemia alone, directly quantifying the accompanying atherogenic dyslipidemia. This approach facilitates a more personalized and pre-emptive management strategy, enabling earlier intensification of lipid-focused therapy among high-risk individuals.

Although previous studies have validated the prognostic value of the Atherogenic Index of Plasma (AIP), most have examined its additive role within broad cardiovascular risk stratification frameworks. The present study provides a more focused perspective by defining the specific clinical context in which AIP demonstrates maximal utility. For instance, while Mora et al. (23) demonstrated an association between AIP and all-cause mortality in the general population, their analysis did not assess its capacity to refine glycemia-based risk classification in T2D. Similarly, Won et al. (15) reported strong associations between TG/HDL-C and insulin resistance but did not evaluate clinically oriented reclassification metrics such as Net Reclassification Improvement (NRI) (24), nor did they address the metabolically ambiguous, early dysglycemic range.

Our findings underscore that AIP is not merely a global cardiovascular risk indicator but rather a clinically actionable decision-support biomarker that significantly enhances the discriminatory performance of a conventional model (HbA1c, age, sex)—particularly within the metabolically decisive 8.0%–8.5% transition zone (categorical NRI = 0.384). In this regard, our study advances existing literature by not only corroborating AIP's prognostic relevance but also delineating the precise clinical scenario in which its strategic application can optimize individualized patient management.

The critical HbA1c range of 8.0%–8.5% identified in our analysis aligns closely with previously reported metabolic transition points in the literature. The ACCORD trial (9) demonstrated a paradoxical increase in cardiovascular mortality with intensive glycemic control (HbA1c < 6.0%), suggesting that less stringent targets around 8.0% may be safer for individuals with high cardiovascular risk. Consistently, the AACE/ACE guidelines (10) recommend more flexible HbA1c goals for patients with advanced T2D or substantial comorbidity burden.

Our findings provide a plausible mechanistic explanation for these clinical observations: the marked acceleration in atherogenic dyslipidemia beyond the 8.0%–8.5% threshold substantiates the hypothesis that lipid-driven cardiovascular risk rises non-linearly once this metabolic tipping point is exceeded. This interpretation is supported by Dullaart et al. (25), who reported impaired HDL functionality at comparable glycemic levels, and by von Eckardstein and Sibler (26), who described similar metabolic breakpoints in the pathogenesis of diabetic dyslipidemia. Collectively, this convergent evidence positions the 8.0%–8.5% HbA1c interval as a critical inflection zone for the emergence of cardiometabolic complications in T2D.

The interpretation of these findings should take into account several limitations. The cross-sectional, single-center design precludes causal inference and may limit the generalizability of the results. Furthermore, the restriction to adults aged 18–65 years, while providing a metabolically more homogeneous cohort, may limit applicability to older populations with T2D. Moreover, as data were retrospectively obtained from electronic health records, potentially important confounders—such as body mass index, dietary habits, physical activity, and alcohol intake—were not available for analysis. Although our statistical models suggest a mechanistic pathway, the cross-sectional nature inherently limits the ability to confirm temporal directionality; therefore, longitudinal studies are required to validate whether therapeutic improvement in HbA1c effectively reverses these specific atherogenic lipid shifts. Despite these limitations, the study possesses several notable strengths that enhance the robustness of its findings. By restricting the cohort to individuals not receiving lipid-lowering therapy, we were able to characterize the natural course of dyslipidemia progression without pharmacological confounding. Additionally, same-day measurement of HbA1c and lipid parameters minimized temporal variability, thereby strengthening internal validity. Most importantly, the HbA1c thresholds and reclassification performance metrics identified here provide a sound basis for hypothesis generation and warrant validation in larger, multicenter, prospective cohorts.

## 5 Conclusions

In summary, this study demonstrates that the relationship between glycemic control and lipid-driven cardiovascular risk in T2D is dynamic and non-linear. We identified the HbA1c range of 8.0%–8.5% as a metabolically critical inflection point characterized by accelerated atherogenic dyslipidemia, thereby providing a plausible pathophysiological explanation for the cardiovascular risk paradox observed in trials such as ACCORD. The predominant mediating role of the TG/HDL-C ratio underscores its value as a mechanistically grounded therapeutic target. Clinically, assessment of AIP or TG/HDL-C in individuals approaching this transition zone may enable earlier recognition of escalating cardiometabolic risk and support more personalized, precision-based management strategies. The compelling implications of these findings warrant confirmation and further exploration in large, prospective, pathophysiologically informed cohorts.

## Data Availability

The raw data supporting the conclusions of this article will be made available by the authors, without undue reservation.
